# Pericoronal Follicles of Asymptomatic Impacted Teeth: A Radiographic, Histomorphologic, and Immunohistochemical Study

**DOI:** 10.1155/2012/935310

**Published:** 2012-01-23

**Authors:** Laura Villalba, Federico Stolbizer, Fabián Blasco, Néstor Raúl Mauriño, María Julia Piloni, Alicia Keszler

**Affiliations:** ^1^Department of Oral Pathology, School of Dentistry, University of Buenos Aires, 1122 Buenos Aires, Argentina; ^2^Department of Buccomaxillofacial Surgery and Traumatology, School of Dentistry, University of Buenos Aires, 1122 Buenos Aires, Argentina

## Abstract

*Objective*. To associate radiographic and histopathological features of pericoronal follicles (PFs) of asymptomatic impacted teeth and evaluate cell proliferation and apoptosis in the epithelium. *Study Design*. Epithelium and mesenchyme of radiographically normal (NPF ≤ 2.5 mm) and hyperplastic (HPF 2.6 to 5 mm) PF (*n* = 140) were studied histologically. Cell proliferation (PI) and epithelial apoptosis were evaluated by Ki-67 and bcl-2 expression in 14 NPFs and 10 dentigerous cysts (DCs). *Results*. Radiographically, 127 were NPFs and 13 were HPFs; 87.8% of total PFs exhibited epithelium on the surface. Reduced enamel epithelium was observed in 78 (61.4%) NPFs and 6 (46.2%) HPFs, squamous metaplasia in 17 (13.4%) NPFs and 4 (30.8%) HPFs, and cystic epithelium in 15 (11.8%) NPFs and 3 (23%) HPFs. Mean PI was 1.97 ± 1.25 and 7.97 ± 1.74 in the epithelial component of NPF and DC, respectively; bcl-2 positive expression was observed in 9 (64.3%) NPFs and 7 (70%) DCs. *Conclusion*. The scant epithelial remnant proliferation could imply low risk for development of odontogenic pathologies in the absence of an additional stimulus.

## 1. Introduction

The primitive dental sac, or dental follicle, which originates from odontogenic ectomesenchyme, is part of the tooth germ and is physiologically involved in the formation of cementum, the periodontal ligament, and alveolar bone. Once the tooth has fully developed inside the jaw, the coronal part of the follicle is termed pericoronal sac or follicle (PF) and occasionally persists adjacent to the crown of unerupted or impacted teeth [1]. It is composed of fibrous connective tissue and frequently contains epithelial residues of odontogenesis, which could be the starting point of pathology [2–4]. Radiographically, it appears as a thin pericoronal radiolucency considered normal by some authors when it is less than 3 mm thick [5, 6] and by others when it is no thicker than 2.5 mm [7]. However, scientific evidence supporting this assumption is limited and there is no internationally accepted consensus on the clinical criteria to differentiate between normal and pathological conditions based on radiographic features [8]. Recent studies have reported pathological changes in PF of up to 2.5 mm [2, 9–13] with frequency varying from 23% [11] to 58.5% [13], particularly associated with the third lower molar. Mesgarzadeh et al. [14] found that 53% of the studied ≤2.9 mm PF specimens had developed pathological changes; moreover, the authors point out that their data are limited since the follicular tissue is frequently discarded following extraction rather than being submitted for histopathological evaluation.

The type of pathology found in the different studies also varies. Whereas some studies [2, 10] have reported dentigerous cysts as the only detected pathologic entity, others have also found odontogenic keratocysts, calcifying odontogenic cysts [11], ameloblastomas [9, 14], myxomas, and odontogenic fibromas [13]. Moreover, Leitner et al. [15] reported a case of low-grade fibrosarcoma in an impacted third molar without any clinical evidence of a follicular lesion.

Cysts and odontogenic tumors derive from epithelial cells in oral tissues such as remnants of odontogenic epithelium in the tooth follicle and the epithelial lining of the oral mucosa [16]. Recent studies provide data on the expression of cell proliferation markers in odontogenic epithelial remnants and epithelium of odontogenic cysts [6, 16–18], and of apoptosis-related factors in PF of lower third molars [6].

The objective of the present study was to carry out a comparative radiographic and histological study of asymptomatic PF associated with different teeth and evaluate the degree of cell proliferation and apoptosis of their epithelial component with the aims of detecting changes suggestive of their potential to develop odontogenic pathologies.

## 2. Materials and Methods

The study sample comprised 140 PF associated with teeth impacted in the jaw and indicated for extraction for orthodontic or preventive purposes and included 121 third molars, 8 canines, 4 premolars, and 7 supernumerary teeth.

 The study was approved by the local ethics committee and informed consent was obtained from all treated patients. Panoramic and periapical radiographs were available in all cases and were used to measure the width of the pericoronal space, which was determined from the half of the mesial, distal, and occlusal surfaces; the widest region was selected [19]. In order to establish comparisons, PFs were divided into two groups according to pericoronal space width: clinically normal (NPF) ≤2.5 mm and hyperplastic (HPF) 2.6 mm to 5 mm. Those presenting continuity with the cortical plate in the radiographic image, a history of pericoronaritis in the region, and/or a change in the overlying lining mucosa were excluded from the study. Removal of the teeth and PF was performed under local anesthesia as an outpatient surgical procedure; 4% carticaine chlorhydrate with 1 : 100000 adrenalin was used. Ostectomy was performed using rotary instruments under copious water irrigation. Odontosection was performed when necessary to facilitate tooth removal. The PFs were removed from the bone bed using atraumatic instruments and were fixed in 10% buffered formalin. The material was embedded in paraffin and 4 *μ*m sections were obtained and stained with hematoxylin eosin for histologic evaluation. Microscopic examination of the epithelial and mesenchymatic components of all the specimens was performed by two calibrated observers. The epithelium on the surface was morphologically classified as reduced enamel epithelium or squamous metaplasia epithelium, and continuous stratified squamous epithelium lining the surface of the PF was considered cystic epithelium [2, 3]. The mesenchymatic component was analyzed to determine the type of connective tissue, that is, loose or dense, the presence or absence of inflammation, and the presence of intramural epithelial cords-islands, and dystrophic calcifications. After performing histomorphologic analysis of the total cases, NPFs were selected to evaluate epithelial cell proliferation and apoptosis. Only NPF exhibiting no aggregate inflammation and sufficient epithelial length to enable assessment of at least 3 randomly selected microscopic fields were studied immunohistochemically. Fourteen NPFs were obtained, 9 of which exhibited reduced enamel epithelium and 5 showed squamous metaplasia and compared with 10 DC.

Sections 4 *μ*m thick were cut and mounted on electrically positively charged slides and dried. Immunohistochemical detection was performed by a standard avidin-biotin peroxidase procedure. After deparaffinization and rehydration, sections were incubated in 0.01 M citrate buffer in a microwave oven for 15 min for antigen retrieval. The slides were then washed in phosphate-buffered saline (PBS) for 30 min at room temperature and incubated in 0.3% H_2_O_2_ in methanol for 10 min to block endogenous peroxidase activity. Primary monoclonal antibody to the cell proliferation-associated antigen Ki-67 (Dako-Denmark) dilution 1 : 100, and monoclonal antibody to the oncoprotein bcl-2 (Novocastra-Leica, New CastleUK) dilution 1 : 100, were applied overnight at 4°C. The sections were subsequently washed in PBS and processed for detection of the positive immunohistochemical reaction using the avidin-biotin peroxidase system (Detection System Vectastain Elit, Vector Laboratories, Burlingame, CA, USA) and diaminobenzidine as the chromogen. Sections were finally counterstained with Harris hematoxylin, cleared, and mounted.

Five samples of healthy gingival mucosa were collected from the same patients while undergoing surgical tooth removal served as control. The total number of cells and number of positively stained cells was counted in three randomly selected fields using a 10 × 10 ocular grid at 40x magnification. The percentage of cells showing positive nuclear staining for Ki-67 was calculated and expressed as a cell proliferation index (PI). The percentage of cases exhibiting cytoplasmic staining for bc1-2 was calculated in each group. Data were tabulated and compared statistically using Student's *t*-test.

## 3. Results

The 140 PF corresponded to 79 patients, 28 male and 51 female. Mean age was 20.01 years with an age range from 9 to 50 years. The patients were medically healthy, had not taken any drugs within 30 days prior to surgery, and showed no changes in the overlying mucosa.

**Figure 1:**
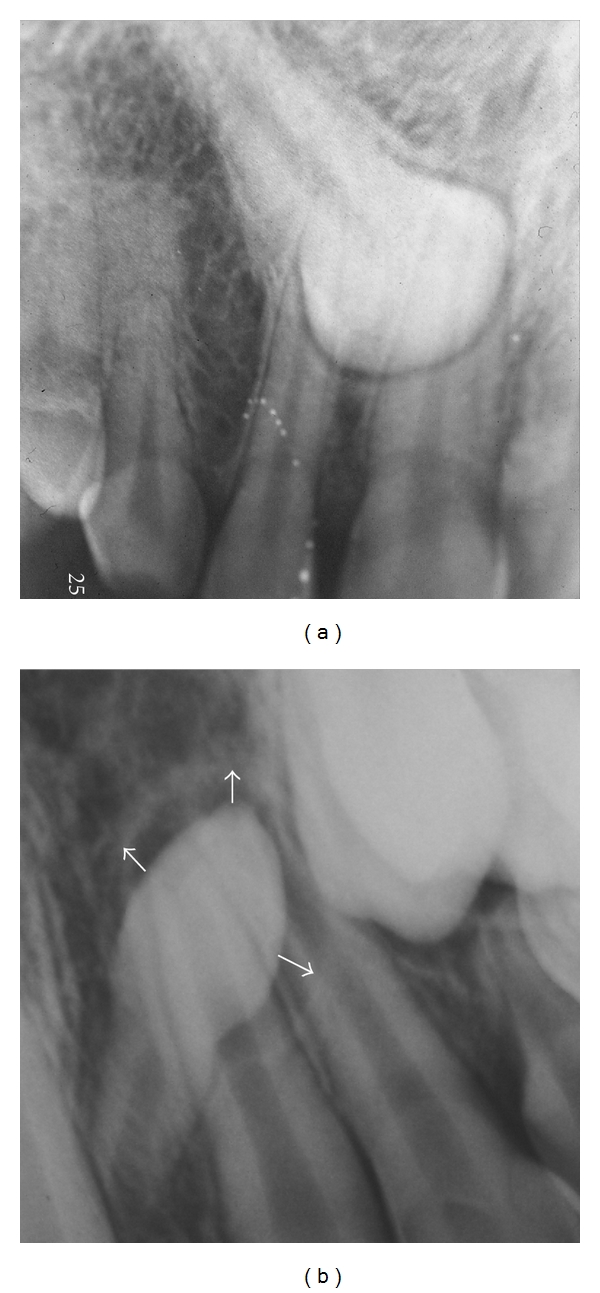
(a) NPF in impacted maxillary canine 14-year-old male. (b) HPF in maxillary supernumerary tooth 9-year-old female.

**Figure 2:**
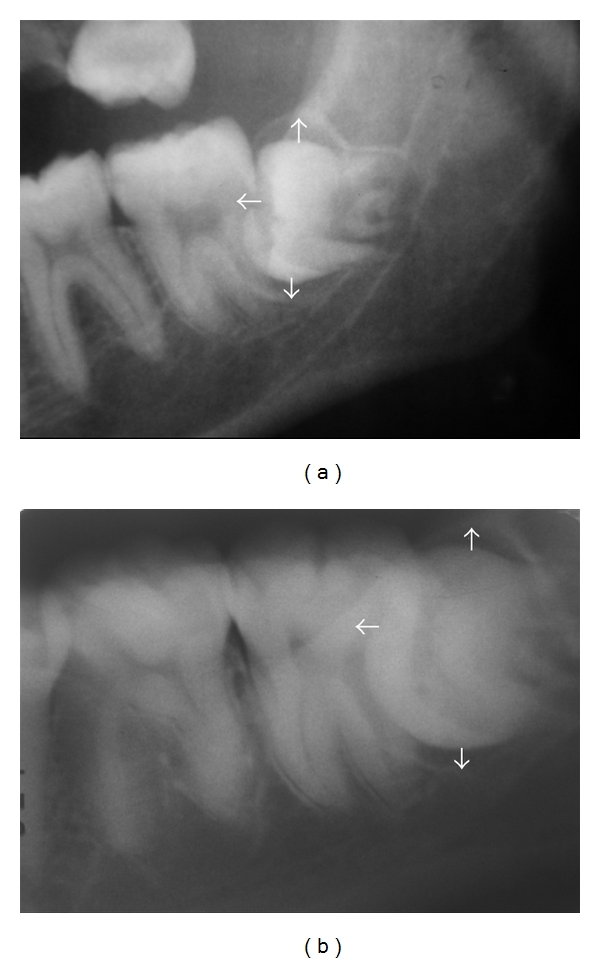
HPF in impacted mandibular third molar; 19-year-old female. (a) Panoramic radiograph. (b) Periapical radiograph of the same case.

According to localization, 72 cases (51.4%) were associated with lower third molars, 49 (35%) with upper third molars, 8 (5.7%) with canines (7 upper and 1 lower), and 4 (2.8%) with upper premolars. The remaining 7 cases (5%) were associated with supernumerary teeth. According to their radiographic dimensions, 127 were NPFs (≤2.5 mm) and 13 were HPFs (2.6 to 5 mm) (Figures 1 and 2).

The general histomorphologic evaluation of the 140 PF showed that 123 of them (87.8%) exhibit epithelium on the surface, which was reduced enamel epithelium in 84 cases, with squamous metaplasia in 21 cases and cystic in the remaining 18. The connective component was dense in 112 of cases, 131 of which presented cords or islands of inactive odontogenic epithelium and 68 cases presented inflammation. The data corresponding to NPF and HPF are shown and compared in Table 1.

**Figure 3:**
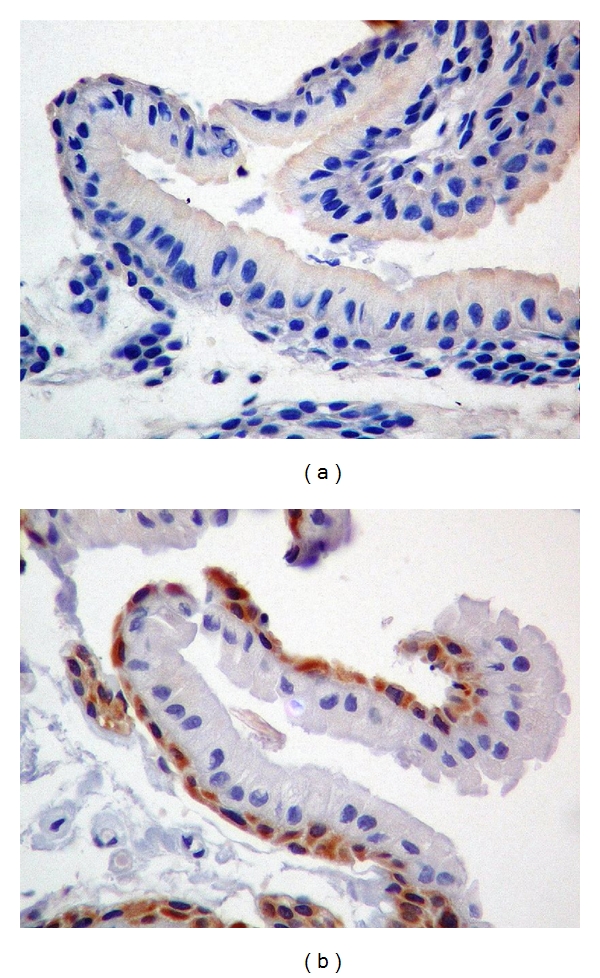
Immunohistochemical features in enamel reduced epithelium of a NPF. Mandibular third molar: 16-year-old female. (a) Negative expression for Ki-67 in nuclei of the epithelial cells. (b) Diffuse expression for bcl-2 in the cytoplasm of epithelial cells. (Original magnification ×40).

**Figure 4:**
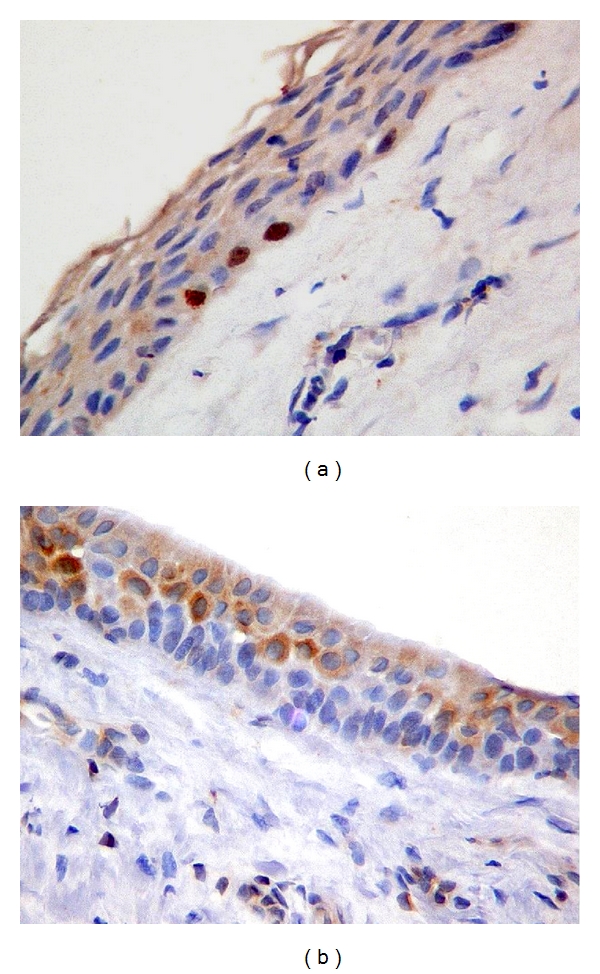
Immunohistochemical features in epithelial lining of DC. Mandibular third molar: 18-year-old female. (a) Ki-67 positive expression in some nuclei of basal cells. (b) Positive staining for bcl-2 throughout the cytoplasm of epithelial cells. (Original magnification ×40).

Immunohistochemical reactivity for Ki-67 and bc1-2 in the epithelial component of the studied NPF and DC is summarized in Table 2. Ninety-three percent of NPF and 100% of DC exhibited immunopositivity for Ki-67 in the nuclei of basal and suprabasal epithelial cells. PI values ranged from 0.85% to 3.12% in NPF and from 4.66% to 11.2% in DC. Mean PI was 1.97 ± 1.41% in the epithelial component of NPF and 7.97 ± 2.05% in the epithelial lining of DC. Positive staining for bcl-2 was detected throughout the cytoplasm of cells in 9 FPN and 7 QD (Figures 3 and 4). The positive ratio of bcl-2 was slightly lower in NPF compared to DC. Ki-67 and bc1-2 expression in each type of epithelium of NPF is shown in Table 3. PI of reduced enamel epithelium and of epithelium with squamous metaplasia was 1.76% and 2.37%, respectively; the difference was not statistically significant. Fifty-five percent of cases with reduced enamel epithelium and 80% of cases with squamous metaplasia were immunoreactive for bc1-2.

## 4. Discussion

The present study showed that most NPF and all HPF exhibited epithelium on the surface. Reduced enamel epithelium was the most frequent type in both groups, though it was more frequent in NPF than in HPF. Squamous metaplasia and cystic epithelium were more frequent in HPF as compared to NPF. The presence of inflammation and dystrophic calcifications was also higher in HPF than in the NPF; and both showed similar frequency of intramural epithelial islands. There were no significant differences among the teeth in any of the analyzed parameters, in spite of their diverse localization.

PI of epithelial lining was lower in NPF as compared with DC, while no difference in positive bcl-2 expression was observed between both groups.

Data on pathological changes in PF are scant. This is due to the fact that following removal of asymptomatic impacted teeth, particularly in the case of lower third molars, the pericoronal tissue is often discarded rather than being submitted for histopathological evaluation [11]. In addition, despite frequent discussion about the indication for prophylactic removal of impacted third molars, there is still no consensus regarding the need for removal in asymptomatic cases. Thus, some dental health professionals favor observation and periodic monitoring and do not perform immediate removal. There is also controversy in the literature about the criteria to establish differential diagnosis between incipient DC and enlarged or HPF. According to some authors, conclusive diagnosis of DC can only be made based on the identification of a pathological cavity between the dental crown and ectomesenchymal portion during surgery, and they further emphasize that differentiation between the two entities cannot be established by histomorphological analysis [20–22]. Other authors posit that differential diagnosis can be made based on criteria identified by the pathologist, mainly the type of epithelium [2, 3, 17]. Whereas some researchers assert that the presence of squamous metaplasia in the lining of PF is not sufficient to diagnose DC [21], others suggest that it is the initial stage of the lesion since it presents greater cell proliferation compared to healthy follicular tissue [17]. In agreement with reports in the literature [13], the results obtained in the present study show that most PF (87.8%) exhibited epithelial lining. Our results differ, however, when considering the type of epithelium. The predominant type of epithelium in the present series was reduced enamel epithelium, though frequency differed between NPF and HPF. Whereas 61.4% of NPF presented reduced enamel epithelium, this percentage dropped to 46.2% in HPF, which showed an increase in squamous metaplasia compared to NPF. The presence of cystic epithelium was also higher in HPF (23%) than in NPF (11.8%). It is noteworthy that these findings are markedly lower than those reported by other authors [2, 9, 10, 13, 17]. The mesenchymatic component was predominantly fibrous dense connective tissue, containing cords or islands of inactive epithelium in most cases and frequently evidencing inflammation, which was greater in HPF. Taking into account the role inflammation may play in the stimulation of epithelial proliferative activity and subsequent changes in PF, evaluation of the proliferation index and apoptosis was performed in the present study in cases presenting no inflammatory infiltrate so as to assess epithelial activity as a potential source of pathology in the absence of an added stimulus. The results obtained herein showed that the percentage of proliferation was low in NPF and markedly lower when compared with DC. Bcl-2 expression was also lower in PF than in DC. These findings differ from those reported by Edamatsu et al. [6] who found a slightly higher number of Ki-67-positive cells in DC than in PF and a significantly lower positive ratio of bc1-2 in PF compared to DC. Results corresponding to enamel-reduced epithelium and squamous metaplasia also differ. In our series of cases, enamel reduced epithelium showed lower cell proliferation index and bc1-2 expression than squamous epithelium; the difference, however, was not statistically significant. The values found in the present study are substantially lower than those reported by the aforementioned authors. These differences are probably due to the inclusion of cases with moderate-to-severe inflammation in their study sample. It has been suggested that the presence of inflammation may be associated with PF enlargement, a process that could result in cystic transformation of the follicle. The results, however, are not conclusive [22].

Data reported to date on the potential proliferation of odontogenic epithelial remnants in the PF are scant and inconsistent. This is a source of controversy regarding indication for preventive surgical removal of impacted teeth.

According to the results obtained in this study, the persistence of epithelial remnants in the PF of asymptomatic impacted teeth would seem to imply low risk of development of odontogenic pathologies, given their sparse proliferative activity in the absence of a superimposed stimulus, as is inflammation.

## Figures and Tables

**Table 1:** Histopathological findings in the epithelial and mesenchymatic components of 140 pericoronal follicles divided in NPF and HPF.

Component	NPF		HPF	
	*N*: 127		*N*: 13	
	*n*	%	*n*	%
Epithelial				
Reduced enamel epithelium	78	61.4	6	46.2
Squamous metaplasia	17	13.4	4	30.8
Cystic epithelium	15	11.8	3	23.0
Absent	17	13.4	0	0
Mesenchymatic				
Dense connective tissue	101	79.5	11	84.6
Loose connective tissue	26	20.5	2	15.4
Presenting inflammation	58	45.7	10	76.9
Epithelial islands	118	92.9	13	100
Dystrophic calcifications	24	18.9	4	30.8

**Table 2:** Immunohistochemical findings of Ki-67 and bcl-2 in epithelial components of pericoronal follicles and epithelial lining of dentigerous cysts.

	PI (%)		bcl-2	
	*N*	Ki-67	−	+
NPF	14	1.97 ± 1.41*	5 (35.7%)	9 (64.3%)**
DC	10	7.97 ± 2.05*	3 (30.0%)	7 (70.0%)**
Control	5	6.88 ± 0.49	5 (100%)	0 (0%)

NPF: normal pericoronal follicle, DC: dentigerous cyst.

*P : 0.05.

**P : 0.8.

**Table 3:** Correlation between histopathological and immunohistochemical findings in epithelial components of pericoronal follicles.

	PI (%)		bcl-2	
	*n*	Ki-67	−	+
REE	9	1.76 ± 1.25*	4 (44.4%)	5 (55.5%)**
SM	5	2.37 ± 1.74*	1 (20.0%)	4 (80.0%)**

REE: reduced enamel epithelium, SM: squamous metaplasia.

*P : 0.5.

**P : 0.7.
